# An Unprecedented Role Reversal: Ground Beetle Larvae (Coleoptera: Carabidae) Lure Amphibians and Prey upon Them

**DOI:** 10.1371/journal.pone.0025161

**Published:** 2011-09-21

**Authors:** Gil Wizen, Avital Gasith

**Affiliations:** Department of Zoology, Tel-Aviv University, Tel-Aviv, Israel; University of Bristol, United Kingdom

## Abstract

Amphibians often feed on beetle larvae, including those of ground beetles (Carabidae). Preliminary reports have detailed an unusual trophic interaction in which, in contrast, larvae of the ground beetle *Epomis* prey upon juvenile and adult amphibians. While it is known that these larvae feed exclusively on amphibians, how the predator-prey encounter occurs to the advantage of the beetle larvae had been unknown to date. Using laboratory observations and controlled experiments, we recorded the feeding behavior of *Epomis* larvae, as well as the behavior of their amphibian prey. Here we reveal that larvae of two species of *Epomis* (*E. circumscriptus* and *E. dejeani*) lure their potential predator, taking advantage of the amphibian's predation behavior. The *Epomis* larva combines a sit-and-wait strategy with unique movements of its antennae and mandibles to draw the attention of the amphibian to the presence of a potential prey. The intensity of this enticement increases with decreasing distance between the larva and the amphibian. When the amphibian attacks, the larva almost always manages to avoid the predator's protracted tongue, exploiting the opportunity to attach itself to the amphibian's body and initiate feeding. Our findings suggest that the trophic interaction between *Epomis* larvae and amphibians is one of the only natural cases of obligatory predator-prey role reversal. Moreover, this interaction involves a small insect larva that successfully lures and preys on a larger vertebrate. Such role reversal is exceptional in the animal world, extending our perspective of co-evolution in the arms race between predator and prey, and suggesting that counterattack defense behavior has evolved into predator-prey role reversal.

## Introduction

Role reversal within predator-prey interactions is a rare phenomenon. Although previous studies [1] reviewed cases of role reversal in which the prey confronts its predator, all those cases involved predators whose predation upon each other was regulated by size [2]–[4] or population density [5], or related to a herbivore prey that killed its predator but did not feed on it [1], [6]. The evolution of role reversal within predator-prey interactions has not been explored.

**Figure 1:**
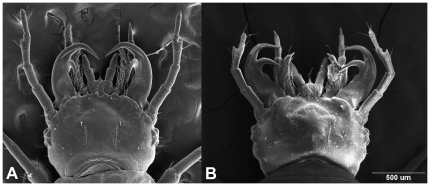
A SEM photograph of larvae heads (first instar) showing specialized double-hooked mandibles. **A** *Epomis dejeani*; **B** *E. circumscriptus*. Scale bar, 500 µm.

Here we present a case in which a predator feeds on a particular group of species, but also becomes a prey of congeners of the latter. Amphibians prey upon a variety of terrestrial arthropods, including ground beetles (Carabidae, [7]–[9]). Adult and larvae ground beetles of the genus *Epomis* (*E. circumscriptus* Duftschmid, 1812 and *E. dejeani* Dejean, 1831) co-occur with amphibians in the same moist habitat [10], [11], sharing the same shelters (e.g. stones, wood debris). This habitat sharing is by no means innocent, because in this unique case it is the adults and larvae of the *Epomis* beetles that prey upon the amphibians which are larger in size [10], [11]. While adult beetles are generalist predators that feed on a variety of food items including amphibians, the larvae are specialists and feed exclusively on amphibians [10]. The larvae feature unique double-hooked mandibles (Figure 1, [12]) that enable them to attach firmly to the amphibian's skin. The larval activity starts soon after onset of terrestrial activity by amphibian metamorphs [10]. Feeding by the young larvae resembles parasitism (sucking body fluids), which is unusual for ground beetle larvae featuring mandibles suitable for cutting and chewing [13]. This parasitic-like feeding often shifts to predation (chewing body tissues) at the later stages, resulting in the amphibian's death [10]. The amphibian may occasionally survive the sucking of body-fluids by the young larva, and in such cases it bears noticeable scars inflicted by the larva's mandibles. A similar interaction was reported for larvae of at least one more species of *Epomis* (*E. nigricans*, [14]–[16]). *Epomis* larvae go through three developmental stages, and at the end of each instar they drop off their amphibian host and molt in a concealed place. After molting the larva seeks a new amphibian host. How this predator-prey encounter evolved to the advantage of the beetle larvae is not known, prompting us to examine this aspect that has shown to be an extraordinary case of role reversal.

## Results

Without exception, in all the observed inter-species interactions the larva showed the same response to the amphibian regardless of the species examined, and the interaction ended in favor of the *Epomis* larva.

**Figure 2:**
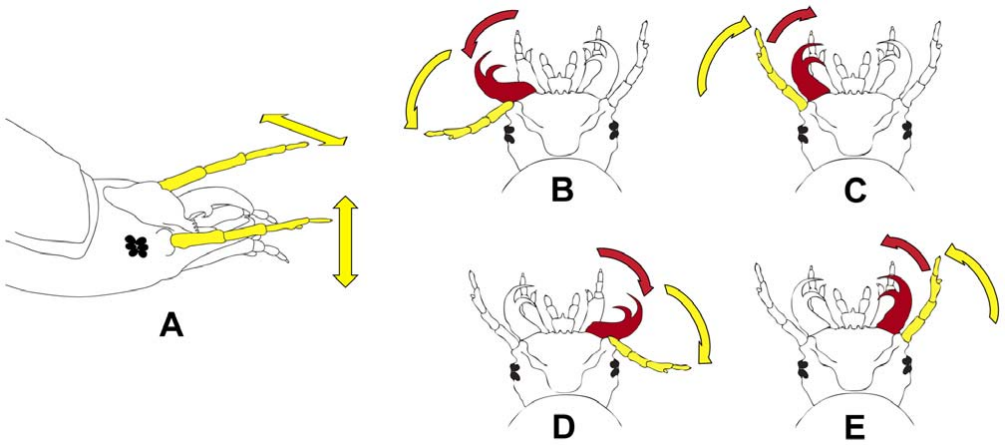
Antennal and mandible movements displayed by *Epomis* larvae during luring. **A** Antennal waving: the antennae (in yellow) move up and down (yellow vertical arrow) and sideways (yellow horizontal arrow) simultaneously; **B** Antennal-mandible cycle starts with an antenna on one side of the head moving sideways (yellow arrow) followed by sideways movement of the mandible (in red) on the same side (red arrow); **C** The mandible and the antenna then move back to their original position (red and yellow arrows); **D** and **E** The cycle is completed by successive identical movements of the antenna and mandible on the other side of the head (yellow and red arrows, respectively).

The experiments revealed that the larvae-amphibian interaction involves an unusual luring behavior displayed by the larva. The luring activity is composed of either antennal waving only or a combination of antennal and mandible movements. In waving, the larva moves its antennae both up and down and sideways (detailed in Figure 2A). The combined antennal and mandible movements can be described as a repeated cycle in which the antenna on one side of the head is moved sideways followed by sideways movement of the mandible on the same side. The mandible and the antenna then move back to their original position. The cycle is completed by successive identical movements of the antenna and mandible on the other side of the head (Figure 2 B–E; Video S1). The larva alternates between waving and the antennal-mandible cycles. The duration of these movements may last for anything from seconds to an hour. Further observations showed that pre- and post-molt larvae that do not feed do not display this behavior.

Larvae of the two *Epomis* species were observed displaying the same luring movements. Moreover, this behavior was not related to a specific larval instar. Shortly after introducing an amphibian into the larva's container, the larva remained in place on the ground and displayed antennal and mandible movements (Figure 2). The amphibian reacted to these movements by approaching the larva (Figure S1), pouncing on it and protracting its tongue in an attempt to seize its apparent prey. The larva responded with a swift head movement towards the pouncing amphibian and, before being grabbed by the latter's tongue, it successfully attached itself to the nearest part of the amphibian's body, mostly the mouth and upper venter areas. Shortly afterwards, the larva repositioned itself on the amphibian's body and initiated feeding (Video S2).

**Figure 3:**
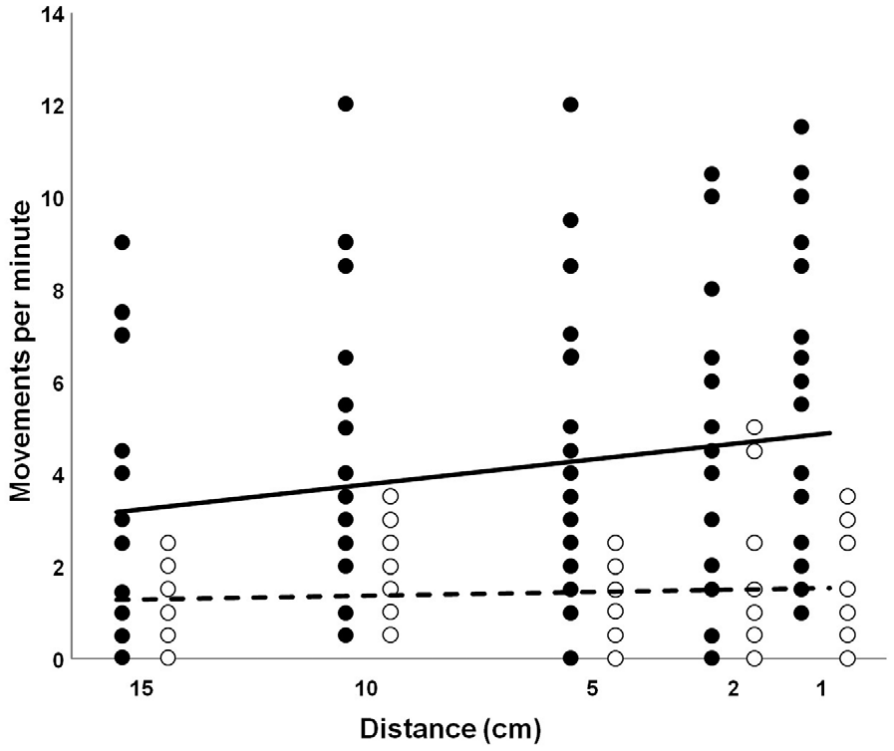
Influence of amphibian presence on the intensity of luring behavior. Luring intensity is expressed as the number of antennal and mandible movements per minute. The test group (n = 23) was exposed to an approaching cage containing a *Pseudepidalea viridis* metamorph. Control group (n = 15) was similarly exposed to an empty cage. As the distance between the amphibian and larva decreased from 15 to 1 cm, the intensity of luring movements in the test group (filled circles) almost doubled (solid line; mean ± SE, 2.8±0.47 and 4.9±0.69 movements/min, respectively). The control group (empty circles) exhibited little change in the intensity of luring movements (dotted line; mean ± SE, 1.1±0.25 and 1.4±0.3 movements/min) in response to the approaching cage. The luring intensity between the two groups was significantly different (p<0.001, F_1,35_ = 39.35, ANCOVA).

We observed that the intensity of the antennal and mandible movements increased with decreasing distance between the amphibian and the *Epomis* larva. In our experiment the intensity of this enticement almost doubled as the distance between the amphibian and the larva decreased from 15 to 1 cm (mean ± SE, 2.8±0.47 and 4.9±0.69 movements/min, respectively; p = 0.053, F_1,35_ = 3.79; Figure 3). The typical antennal and mandible movements were also recorded in the absence of an amphibian; however, their intensity was three times lower when compared to the test group (p<0.001, F_1,35_ = 39.35; Figure 3). In addition, the control group exhibited little change in the intensity of luring movements in response to the approaching cage (mean ± SE, 1.1±0.25 and 1.4±0.3 movements/min; p = 0.274, F_1,35_ = 1.20; Figure 3).

In 70% of the experiments in which an amphibian was introduced into the container with either of the two *Epomis* species larva (n = 382), luring behavior was recorded and the amphibian was consumed and killed by the larva, leaving behind only a pile of bones (Figure S2). In the remaining cases, in which the predator and prey accidentally encountered one another, luring behavior was not displayed by the larva (Video S3) but the interaction ended with the same fatal consequences for the amphibian.

In seven instances the amphibian gulped the larva but then quickly regurgitated it, and the larva was then able to attach itself successfully to the amphibian's skin (in the mouth area; Figure S3 and Video S3). In one additional case, a *P. viridis* metamorph successfully swallowed an *E. circumscriptus* larva, but after ca. two hours during which the larva still survived and moved inside the amphibian's stomach, it was regurgitated (Video S4). This larva, which proved unharmed, then successfully attached itself to the amphibian's body and initiated feeding. As in all other cases, this interaction eventually ended with the amphibian consumed by the larva.

## Discussion

Organisms interact in a variety of ways, one of which is through food consumption as in predator-prey interaction. In most cases in the animal world (90%) it is the larger predator that consumes the smaller prey [17], [18]. Predators have evolved various mechanisms by which to catch their prey and the prey have developed mechanisms to avoid the predator [19]. In insects, for example, predator avoidance includes morphological (e.g. camouflage, warning colors and mimicry), physiological (e.g. chemical defense), and behavioral adaptations (e.g. aggregation, avoidance and counterattack; reviewed in [20] and [7]).

An extremely rare anti-predator behavior is that of role reversal. Up until now, role reversal has been attributed to cases in which a prey actively confronts its predator. In these interactions role reversal either ended with no feeding by either side [1], [6] or involved cases of competition between the predators, in which the larger organism preyed on the smaller one [2]–[4]. Here, we limit the definition of role reversal to cases in which the usual prey becomes the predator, feeding on its potential predator. In the case of the *Epomis* beetle, the larva can be much smaller than its potential amphibian predator (Table S2).

Other than the case of the *Epomis* larvae and amphibians that we report here, we know of only one other example of role reversal that apparently matches our definition. This was demonstrated for rock lobsters (*Jasus lalandii*) that normally consume settling mussels and also prey on whelks (*Burnupena* spp., [5]). Rock lobsters that were transferred to a different location, where whelks occur in high densities, were overwhelmed and consumed by the latter, reversing the normal predator-prey interaction between the two species. This reported case is an exception to the normal lobster-whelk interaction, and occurred as a result of manipulation [5]. In contrast, the *Epomis*-amphibian interaction that we present here is natural and would appear to be the rule in the interaction between them. This suggestion is also supported by several reports of *Epomis* larvae that were found attached to amphibians in the field [10], [14], [16]. In this respect the *Epomis*-amphibian interaction is an exceptional case of role reversal in the animal world. Moreover, unlike any other reported interaction of role reversal, in this interaction the beetle larvae have developed a specific luring behavior to entice amphibians that otherwise regularly prey upon beetle larvae [7]–[9]. Amphibians make predatory decisions relying primarily on prey movement and secondarily on prey size, with prey color being less important [21], [22]. They respond to moving objects in two ways: small moving objects elicit the orientation of the amphibian towards the object and trigger predation; while large moving objects trigger avoidance behavior [23]–[25]. The size of beetle larvae fits an amphibian prey model, making the *Epomis* larva a suitable prey. *Epomis* belongs to the Chlaeniini tribe within the ground beetles, whose larvae are typical surface runners [26] and their movements provoke amphibian predation. We found remains of Chlaeniini larvae related to *Epomis* in amphibian feces that can serve as evidence for such predation. In contrast, larvae of *Epomis* beetles are sit-and-wait predators and as such are not expected to attract amphibians. Luring behavior by means of movement of the larva's antennae and mandibles has apparently evolved to trigger amphibian predation behavior, and this can explain why luring intensifies in the presence of an amphibian. The sit-and-wait strategy conceals the larva but also enables the larva to be ready to respond to the fast approaching amphibian. To avoid being gulped by the amphibian, the beetle larva must overcome its swift charge. For example, the duration of mouth opening and tongue protraction in the marine toad (*Bufo marinus*) is 109±5 ms [27]. Even though the studied larvae remained still and moved only their antennae and mandibles, they succeeded in preying upon the different amphibian species in 100% of the observed interactions. Some amphibians displayed toe-waving upon noticing the *Epomis* larva (Video S2). Such behavior has been attributed to prey luring by amphibians [28]. However, in the case of *Epomis* the larva remained in position and we have no evidence for concluding that it responds to the amphibian's approach only, to the amphibian's toe movement or to both. In a few cases in which the amphibian initially succeeded in capturing the larva, it immediately released it, and the larva eventually initiated feeding on the amphibian. In one extraordinary case the amphibian ingested the larva for ca. 2 hrs before eventually regurgitating it, and the unharmed larva immediately demonstrated its unaffected feeding potency. It should be noted that unlike other sit-and-wait predators that orient themselves in response to the prey's position and movements and only then capture the prey [29], the *Epomis* larvae strike from any position, whether facing the approaching amphibian or not.

Amphibians portray an array of anti-predator responses (e.g. camouflage, warning colors, toxicity, and various behavior patterns, [30]–[33]), but have apparently failed to identify the *Epomis* larvae as dangerous predators. The reason for this may derive from the fact that this trophic interaction is extremely rare relative to the amphibians' successful interactions with *Epomis* congeners as well as from the extremely high rate of predation success on the part of the *Epomis* larvae. Moreover, the strong response of amphibians to small moving objects is probably an inherent handicap, hindering development of a specific avoidance response to the luring behavior displayed by the beetle larvae. To the best of our knowledge the case of *Epomis* larvae and amphibians is the only known natural case of obligatory predator-prey role reversal that involves luring behavior. The mechanism of the larva's swift counterattack against the pouncing amphibian is still unknown. How a single insect genus evolved a unique role reversal trophic interaction is currently an enigma. It is possible that the role reversal trophic interaction displayed by *Epomis* larvae evolved as an extreme form of defense against amphibians, a major predator of beetle larvae [7]–[9]. Insects show an array of defenses against predators [20], one form of which is counterattack, in which the prey turns against its predator. This behavior is found mostly in social insects. Although there have been some reports of this behavior in nonsocial insects, those reports involve gregarious insects only, which apparently compensate for their small size in comparison to their predator by being numerous [20]. It is possible that counterattack has also evolved in solitary insects. This could have led to the development of a trophic role reversal with a specialized diet based on the availability of a rich food resource provided by the larger prey. Specialized diet can in turn, act as an evolutionarily selective force for the development of a luring behavior.

## Materials and Methods

Larvae of two *Epomis* species (*Epomis dejeani* and *Epomis circumscriptus*) were obtained *ex-ovo* in the laboratory from beetles collected in the wild. A total of 420 larvae were used in this study. The larvae were kept in 0.5 liter plastic containers (7.5 cm high; 10.5 cm diameter) with moist peat as substrate. Three species of amphibians, *Pseudepidalea viridis* (Anura: Bufonidae), *Hyla savignyi* (Anura: Hylidae) and *Pelophylax bedriagae* (Anura: Ranidae), were collected as tadpoles from drying rain-pools, and were kept in containers until completion of their metamorphosis. Two additional species, *Ommatotriton vittatus* and *Salamandra infraimmaculata* (Caudata: Salamandridae), are rare and therefore only a few specimens were used in this study. The amphibians' containers measured 21×11×15 cm and contained moist peat as substrate. A piece of wood bark was placed in the container as shelter for the amphibian metamorphs. Each container housed 5 specimens of the same amphibian species. The amphibians were fed regularly with house crickets (*Acheta domestica*) except for the day of the experiment. For the experiments we used amphibians that were two-three weeks post-metamorphosis, corresponding to the size of juvenile amphibians encountered by *Epomis* larvae in the field (Table S1). All animals were kept indoors in a room under constant temperature (25°C±1°C).

The larva's mandibles were photographed under a JEOL 840A SEM at 15 kV (X 50–80).

We conducted observations in order to record the response of *Epomis* larvae to different amphibian species at the moment of encounter. Larva-amphibian encounter observations were conducted under the same indoors conditions described above. We used one liter plastic containers (10.5 cm high; 14.5 cm diameter) with moist peat as substrate. A randomly selected naive metamorph of a known amphibian species was introduced into a container with a naive, two days post-molt *Epomis* larva. This was repeated with the different amphibian species (anurans: *P. viridis, H. savignyi* and *P. bedriagae*; caudatans: *O. vittatus* and *S. infraimmaculata*) and beetle species (*E. dejeani* and *E. circumscriptus*), in a total of 382 trials (Table 1). Each specimen of larva and amphibian was used only once. All larvae used in the experiments were at the same level of starvation. Similarly, all the amphibian specimens used in the experiments were at the same level of starvation. We documented the feeding interaction using video clips and still photographs (Canon powershot SX10 video camera, and DSLR, Canon EOS 20D and Canon EOS 50D, respectively). The video recording started 10 seconds before introducing the amphibian into the container with the beetle larva, and was carried out in 10 minute clips until the larva had attached itself to the amphibian's body and started feeding.

**Table 1:** Experimental design of larva-amphibian encounters for *Epomis circumscriptus* and *Epomis dejeani* larvae involving different amphibian species.

	*Epomis circumscriptus*			*Epomis dejeani*			
Amphibian species	1^st^ instar	2^nd^ instar	3^rd^ instar	1^st^ instar	2^nd^ instar	3^rd^ instar	Total
*Pseudepidalea viridis*	20	21	63	21	22	67	214
*Hyla savignyi*	17	16	40	9	18	12	112
*Pelophylax bedriagae*	4	7	7	3	3	5	29
*Ommatotriton vittatus*	2	3	3	2	3	4	17
*Salamandra infraimmaculata*	3	2	2	1	1	1	10
Total	46	49	115	36	47	89	

For examination of the luring behavior a naive, third instar (two days post-molt) larva of *E. circumscriptus* was placed in a 20×20×6 cm container with moist peat as substrate. After acclimation for 90 minutes, a naive metamorph of the green toad (*P. viridis*) enclosed within a 4×4×4 cm netted cage was introduced into the container. The cage with the metamorph was gradually moved on a track towards the larva, reducing the distance between them from 15 to 10, 5, 2 and 1 cm. It was maintained at each distance point for two minutes to record the intensity of the larva's antennal and mandible movements. All larvae used in this experiment were at the same level of starvation and each larva was used only once. The test group (n = 23) was exposed to an approaching cage containing the *P. viridis* metamorph. A control group (n = 15) was similarly exposed to an empty cage. We started the experiments with the control, using an empty cage, thus eliminating any hidden amphibian-related factor that might influence the behavior of this group. The larva's intensity of enticement in response to the approaching cage was expressed as the number of antennal and mandible movements per minute. Analysis of covariance (ANCOVA) was used to examine the differences in luring intensity between the control and the test group with the distance from the cage as the covariant. Raw data were non-normal and therefore were transformed using Box-Cox in order to apply the analysis. The statistical analysis was performed using Statistica ver. 8 (StatSoft, Inc).

## Supporting Information

Figure S1 ***Pseudepidalea viridis*** **metamorph attracted to a larva of** ***Epomis circumscriptus*** **that displays antennal movements.**
(TIF)

Figure S2 **Remains of** ***Pseudepidalea viridis*** **metamorph left after the amphibian has been consumed by a larva of** ***Epomis dejeani*** **.**
(TIF)

Figure S3 **Larva of** ***Epomis circumscriptus*** **attached to the mouth of** ***Pseudepidalea viridis*** **metamorph.**
(TIF)

Table S1 **Weight and length of juveniles of five amphibian species used in the experiments.**
(DOC)

Table S2 **Range and mean (±SE) of body length (mm) of** ***Epomis*** **larvae used in the experiments.**
(DOC)

Video S1 ***Epomis circumscriptus*** **larva displays luring movements, combined movements of antennae and mouthparts.**
(AVI)

Video S2 **A** ***Pseudepidalea viridis*** **metamorph is attracted and lured to an ambushing** ***Epomis dejeani*** **larva.** The amphibian can seen displaying toe-waving before pouncing on the larva.
(AVI)

Video S3 **A** ***Pelophylax bedriagae*** **metamorph successfully gulps an** ***Epomis circumscriptus*** **larva but eventually tries to get rid of it.** It fails because the larva has already firmly attached itself to the side of the amphibian's mouth.
(AVI)

Video S4 **A rare case where a** ***Pseudepidalea viridis*** **metamorph successfully swallowed an** ***Epomis circumscriptus*** **larva, but after ca. two hours, regurgitated it and was ultimately consumed by the larva.** In the beginning of this interaction the amphibian failed to eject the larva from its mouth despite repeated efforts. The larva's body, which was partly outside the amphibian's mouth, was stained with blood, evidence of injury inflicted upon the amphibian. Eventually the larva was swallowed completely and was seen moving inside the amphibian's stomach until it was finally regurgitated two hours later. The larva was covered with mucus, positioned sideways and motionless, but otherwise seemed unharmed. When the amphibian moved and was positioned above the motionless larva, the latter suddenly responded by springing to life, grasping the amphibian's venter.
(AVI)
