# Supplementary figures and images for: An Unprecedented Role Reversal: Ground Beetle Larvae (Coleoptera: Carabidae) Lure Amphibians and Prey upon Them
Source: PLoS One. 2011 Sep 21;6(9):e25161. doi: 10.1371/journal.pone.0025161 (PMC3177849; doi:10.1371/journal.pone.0025161)

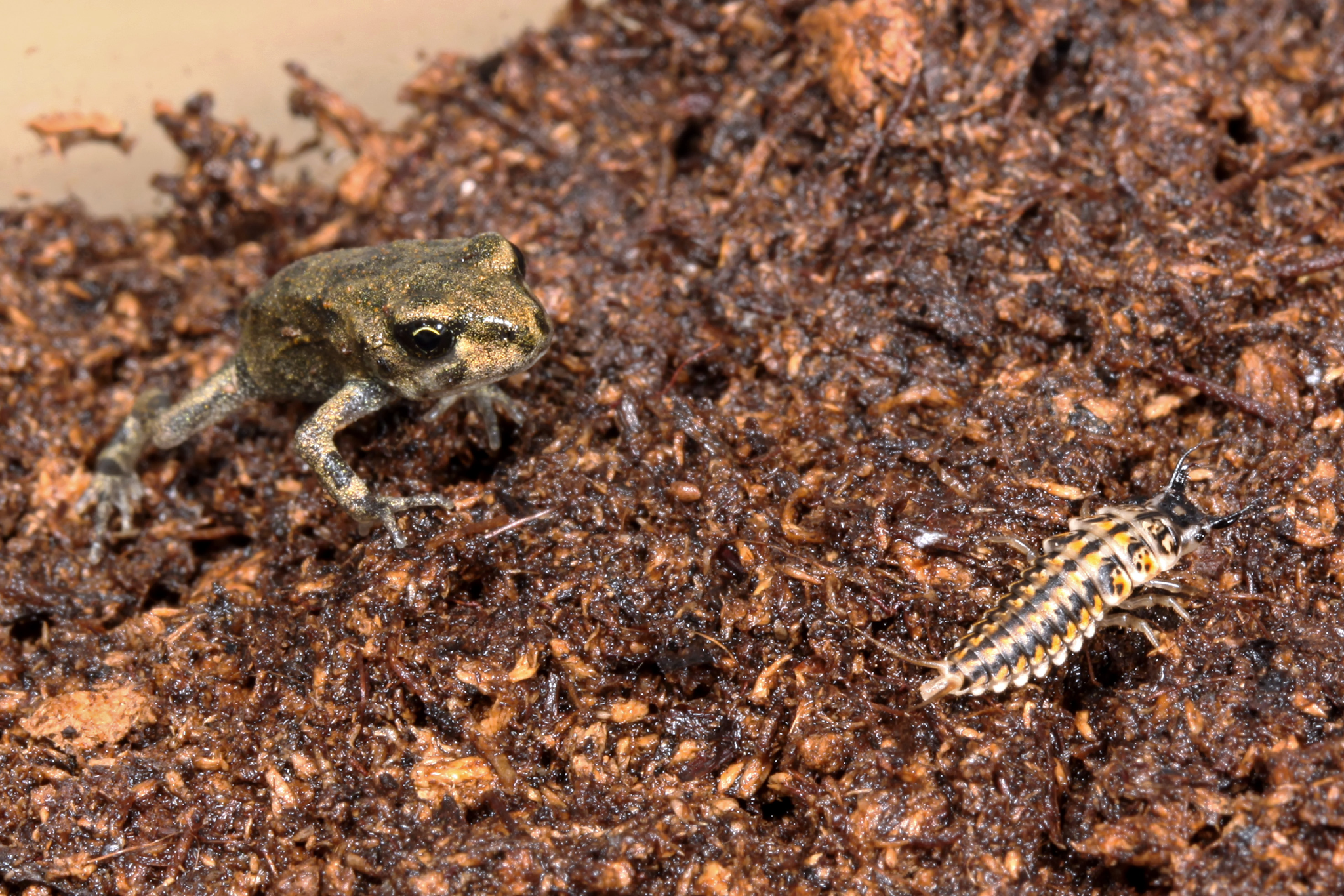

Supplement: Figure S1 — Pseudepidalea viridis metamorph attracted to a larva of Epomis circumscriptus that displays antennal movements. (TIF) [file pone.0025161.s001.tif]

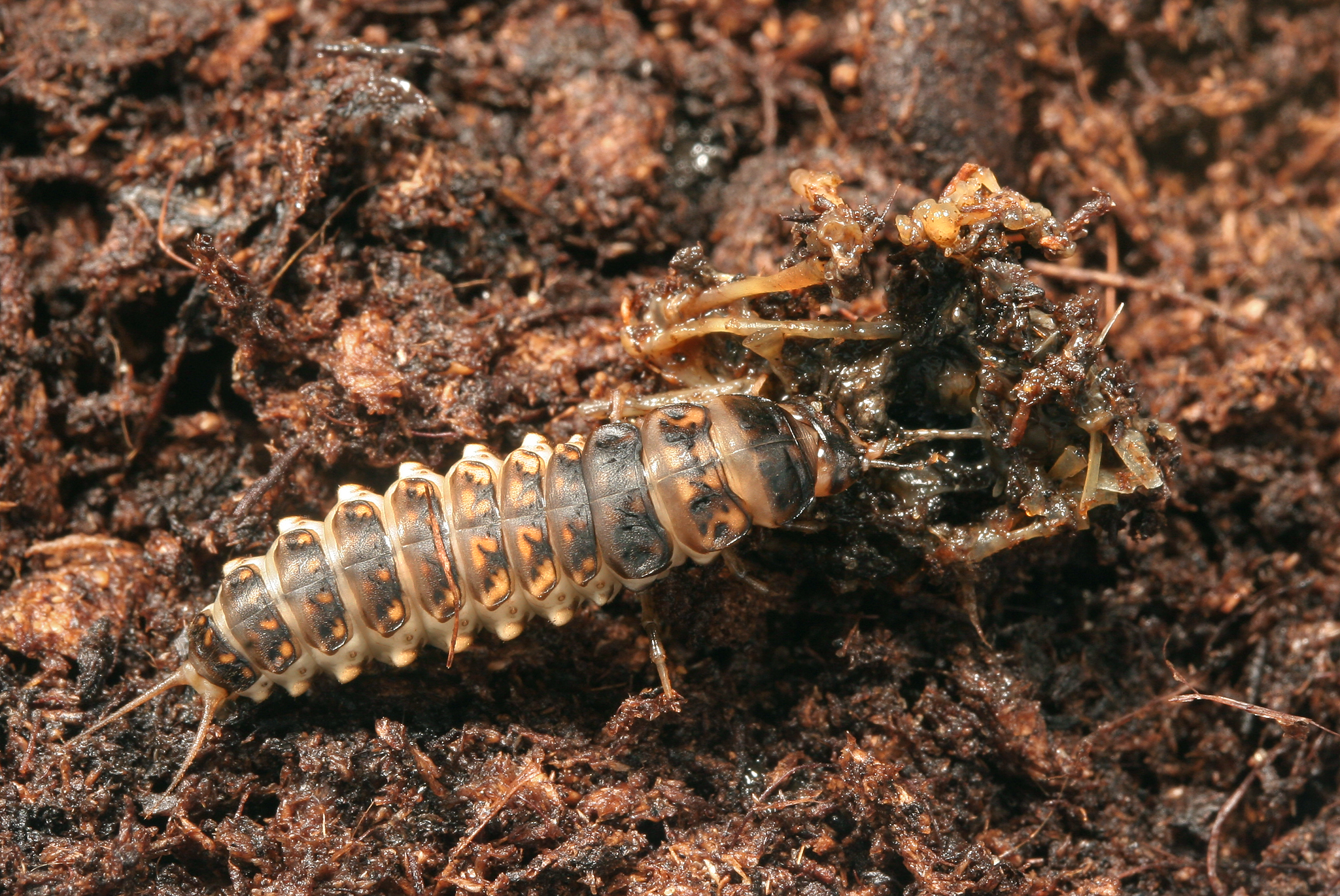

Supplement: Figure S2 — Remains of Pseudepidalea viridis metamorph left after the amphibian has been consumed by a larva of Epomis dejeani . (TIF) [file pone.0025161.s002.tif]

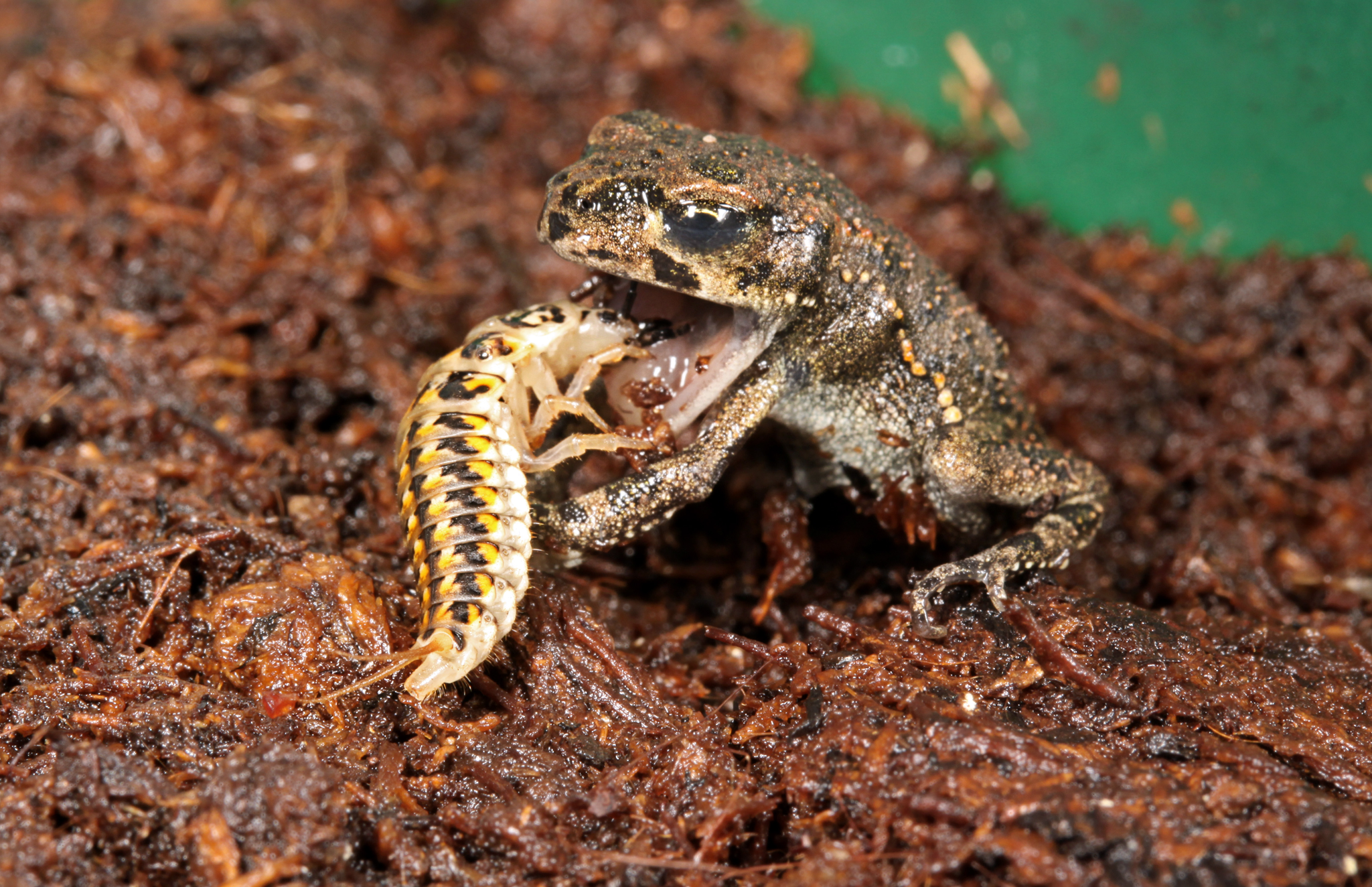

Supplement: Figure S3 — Larva of Epomis circumscriptus attached to the mouth of Pseudepidalea viridis metamorph. (TIF) [file pone.0025161.s003.tif]
