# Supplementary material for: An Unprecedented Role Reversal: Ground Beetle Larvae (Coleoptera: Carabidae) Lure Amphibians and Prey upon Them
Source: PLoS One. 2011 Sep 21;6(9):e25161. doi: 10.1371/journal.pone.0025161 (PMC3177849; doi:10.1371/journal.pone.0025161)
Supplement: Table S1 — Weight and length of juveniles of five amphibian species used in the experiments. (DOC) [file pone.0025161.s004.doc]

**Table S1.** Weight and length of juveniles of five amphibian species used in the experiments^a^.

|  | **Weight (g)** | | **Length (mm)** | |  |
| --- | --- | --- | --- | --- | --- |
| **Amphibian species** | **Mean ±SE** | **Range** | **Mean ±SE** | **Range** | **n** |
| *Pseudepidalea viridis* | 0.38±0.03 | 0.25-0.66 | 16.3±0.38 | 14-18.5 | 214 |
| *Hyla savignyi* | 0.24±0.01 | 0.17-0.3 | 15.8±0.31 | 14-17.1 | 112 |
| *Pelophylax bedriagae* | 1.24±0.14 | 0.82-1.65 | 23.4±0.61 | 22-25.5 | 29 |
| *Ommatotriton vittatus* | 0.21±0.01 | 0.17-0.24 | 33.0±0.83 | 31-36 | 17 |
| *Salamandra infraimmaculata* | 1.19±0.16 | 0.72-1.67 | 54.7±1.85 | 48.5-60 | 10 |

**^a^** Length measurements (snout-vent for anurans; snout-end of tail for urodales) were taken with a caliper (± 0.05mm) and weight measurements using analytical scale (± 0.001g). n indicates number of specimens.
