# Supplementary material for: An Unprecedented Role Reversal: Ground Beetle Larvae (Coleoptera: Carabidae) Lure Amphibians and Prey upon Them
Source: PLoS One. 2011 Sep 21;6(9):e25161. doi: 10.1371/journal.pone.0025161 (PMC3177849; doi:10.1371/journal.pone.0025161)
Supplement: Table S2 — Range and mean (±SE) of body length (mm) of Epomis larvae used in the experiments. (DOC) [file pone.0025161.s005.doc]

**Table S2.** Range and mean (±SE) of body length (mm) of *Epomis* larvae used in the experiments^a^.

|  | **1^st^ instar** | | **2^nd^ instar** | | **3^rd^ instar** | |
| --- | --- | --- | --- | --- | --- | --- |
| ***Epomis* species** | **Mean ±SE** | **Range** | **Mean ±SE** | **Range** | **Mean ±SE** | **Range** |
| *Epomis dejeani* | 4.7±0.07 (36) | 4.4-5 | 8.4±0.11 (47) | 8-9.1 | 13.6±0.29 (89) | 12.1-15.1 |
| *Epomis circumscriptus* | 4.9±0.04 (46) | 4.7-5.1 | 8.8±0.15 (49) | 8-9.4 | 13.9±0.24 (115) | 11.9-16.1 |

**^a^** Length measurements (from the tip of the mandible to the end of the abdomen) were taken with a caliper (± 0.05mm). Number in parentheses indicates number of specimens.
